# Probing Proteoform Heterogeneity From Single Human Oocytes

**DOI:** 10.1016/j.mcpro.2026.101566

**Published:** 2026-04-09

**Authors:** Nickolas P. Fisher, Vijaya Lakshmi Kanchustambham, Elizabeth L. Tsui, Chelsea Lock, Tian Xu, Hannah B. McDowell, Indira Pla, Diane C. Saunders, Jared O. Kafader, Monica M. Laronda, Neil L. Kelleher

**Affiliations:** 1 Department of Chemistry and Molecular Biosciences, Chemistry of Life Processes Institute, Proteomics Center Excellence, Northwestern University, Evanston, Illinois, USA; 2 Stanley Manne Children’s Research Institute, Ann & Robert H. Lurie Children’s Hospital of Chicago, Chicago, Illinois, USA; 3 Department of Pediatrics, Feinberg School of Medicine, Northwestern University, Chicago, Illinois, USA; 4 Department of Obstetrics and Gynecology, Feinberg School of Medicine, Northwestern University, Chicago, Illinois, USA; 5 Chan Zuckerberg Biohub Chicago, Chicago, Illinois, USA

**Keywords:** oocytes, top-down mass spectrometry, posttranslational modifications, proteoforms, single-cell proteomics

## Abstract

Ovarian tissue cryopreservation is a fertility preservation strategy available to prepubertal patients undergoing gonadotoxic treatment who are at risk of developing premature ovarian insufficiency or for those whose treatment cannot be delayed. While cryopreserved tissue can be utilized for ovarian tissue transplantation to restore hormone function and fertility in some of these individuals, those with a high risk of malignant cell reintroduction currently have no options. *In vitro* maturation (IVM) of isolated immature oocytes from prepubertal patients is not yet successful enough for the clinic, necessitating study of the molecular factors influencing oocyte quality for IVM success. In this study, we characterized intact proteoforms from single human oocytes obtained from ovarian tissue cryopreservation and tissue donation in a donor cohort aged 2 to 33 years old to identify changes in the oocyte proteome across the pubertal transition. Utilizing single-cell proteoform imaging mass spectrometry (scPiMS), which employs a sampling probe to raster over single cells coupled to individual ion mass spectrometry detection, we identified 559 proteins and 769 unique proteoforms across 28 oocytes from eight donors, with an average of 78 unique proteoforms per oocyte. We used scPiMS to selectively sample oocytes and cumulus granulosa cells from single cumulus oocyte complexes to identify proteoforms specific to these cell types. Finally, we determined proteoform landscapes for members of the oocyte-specific subcortical maternal complex (SCMC), KHDC3, and OOEP, and found new proteoforms that differ with donor age. Together, these first-in-class observations provide a foundation for understanding protein-level changes in oocyte biology across puberty to ultimately improve the efficiency of IVM and make fertility restoration options accessible for more patients.

## Highlights

• First direct detection of intact proteoforms from single human oocytes.
• High spatial resolution for selective sampling of oocytes and cumulus granulosa cells.
• Protein heterogeneity between donors and multiple oocytes from a single donor.
• OOEP proteoforms correlate with increasing age; KHDC3 proteoforms are consistent.

## Graphical Abstract

**Figure:**
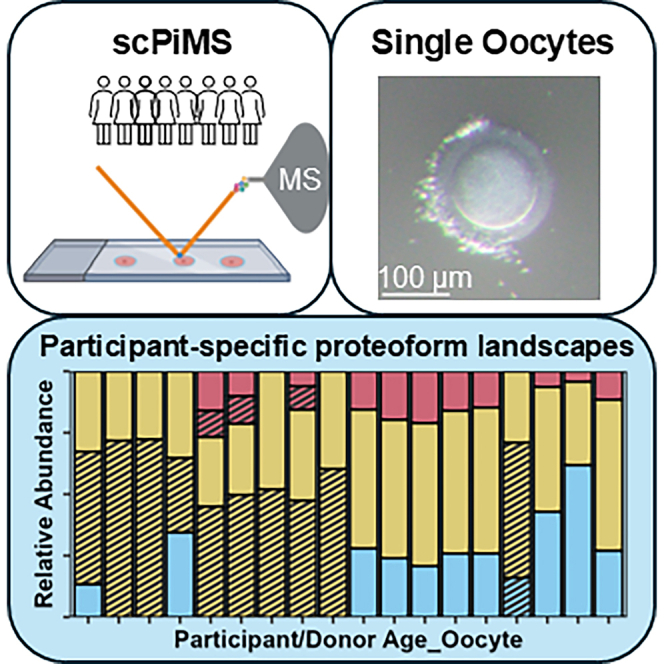

## In Brief

Herein, we report the first proteoform-level detection directly from single human oocytes. Obtained during ovarian tissue processing for cryopreservation for participants ranging in age from 2 to 33 years, we identify oocyte-specific proteoforms and compare how oocyte proteoform landscapes vary among participant specimens, within multiple oocytes from the same donor and across the understudied pubertal transition. We also demonstrate how two key oocyte proteins and their associated proteoforms differ with age.

Human oocytes are generated during fetal gestation and form functional tissue units called follicles that grow and ultimately result in ovulation, enabling fertilization to achieve reproduction. The ovarian reserve, or finite population of follicles, gradually decreases throughout the lifespan until it is mostly depleted around the time of menopause (1). Girls and women who require gonadotoxic treatments often utilize fertility preservation strategies to have options for future biological offspring (2, 3, 4, 5). Ovarian tissue cryopreservation (OTC), a procedure that preserves ovarian tissue containing follicles, is often utilized for prepubertal patients who cannot undergo egg retrieval and are at risk of developing premature ovarian insufficiency or for those whom gonadotoxic therapy cannot be delayed (5, 6, 7, 8). Although implantation of cryopreserved ovarian tissue (ovarian tissue transplantation) post recovery can induce puberty (8, 9) and restore fertility (10, 11, 12), tissue implantation is not available to patients with certain diagnoses that carry the risk of reintroducing malignant cells (13). For these individuals, alternate fertility and hormone restoration strategies such as *in vitro* maturation (IVM) from oocytes released during OTC processing is one option. However, only 38.3% of oocytes released during OTC processing matured by IVM for patients aged 18 to 24 years, only 23.8% developed from patients aged 6 years to the time of menarche and only 4.6% of oocytes from patients aged 1 to 5 years developed into eggs (14). In a similar study of eggs matured from oocyte IVM, the peak quality, or euploid rate, of these eggs was ∼70% and occurred at ages 21 to 32 years old (15). Unfortunately, IVM that generates enough euploid eggs needed for future *in vitro* fertilization (IVF) for prepubertal patients is nearly impossible, leaving these patients who are not candidates for ovarian tissue transplantation with no clinical options to restore fertility (16). The reduced efficiency of IVM for oocytes from young children and adolescents necessitates further investigation of the molecular factors affecting oocyte quality and IVM performance to enhance fertility preservation efforts for these age groups.

The success rate of IVM of immature oocytes at the other end of the age spectrum also varies from 38.3% down to 8.9% with increasing age up to 35 years old (14); several studies have focused on characterizing the proteomic changes in oocytes at various maturation steps and across different age groups. Initial bottom-up proteomics experiments demonstrated the feasibility of performing measurements on single human oocytes and made initial identifications of key regulatory proteins affecting oocyte maturation (17). Subsequent studies elucidated the proteomic differences between oocytes matured *in vivo* via ovarian hyperstimulation and oocytes matured *in vitro*, ultimately identifying higher degrees of heterogeneity in IVM oocytes (18). More recent work has focused on characterizing changes in the oocyte proteome between young and advanced maternal age women, which revealed alterations to the expression of several meiosis proteins as well as a decline in proteasome and protein chaperone expression with age (19). Though these studies have been foundational for our understanding of molecular changes to the oocyte proteome during maturation and aging, they have primarily utilized oocytes from adult patients, leaving proteomic changes in single oocytes from young girls and adolescents uncharacterized (17, 18, 19). In addition, previous studies utilized peptide level information and lack proteoform resolution (20), precluding our understanding of what combinatorial posttranslational modifications and genetic variations may contribute to the heterogeneity in IVM success.

Although analyzing proteoforms in single cells has been challenging due to the reduced sensitivity of intact proteins relative to that of peptides (21), it has been achieved through optimized sample preparation and instrumentation using capillary electrophoresis (22) or nano-desorption electrospray ionization (nano-DESI) mass spectrometry (23, 24). Nano-DESI profiling of intact proteoforms was originally developed for tissue imaging and was initially limited to small proteoforms (<20 kDa) (25, 26). The mass range for nano-DESI analysis of intact proteoforms from tissue was later extended through combination with individual ion mass spectrometry (I^2^MS) detection (27), enabling the identification of hundreds of proteoforms of <70 kDa using this proteoform imaging mass spectrometry (PiMS) technology (28, 29). I^2^MS is an Orbitrap-based charge detection mass spectrometry (MS) technique that detects single proteoform ions and determines ion charge, enabling a >500x increase in sensitivity and a 10x increase in resolving power for intact proteoforms or proteoform fragments over traditional ensemble MS approaches (30, 31). Using PiMS analysis for single cells (scPiMS) allows for the identification of biologically relevant proteoforms in dissociated cells and was validated using rat hippocampus, where cell type was accurately determined for >5000 cells (24).

In this study, we applied scPiMS to single human oocytes isolated during the OTC process from prepubertal, adolescent, and adult participants to identify key proteoforms associated with oocyte function. We used this method to examine the latent heterogeneity of oocyte populations in participants across an age range previously uncharacterized at the single oocyte level and identified diverse proteoforms of key members of the oocyte-specific subcortical maternal complex (SCMC).

## Experimental Procedures

### Ethical Approval for the Collection and Use of Human Tissue

The collection of human ovarian tissue was approved by the Institutional Review Board of Ann & Robert H. Lurie Children’s Hospital (prepubertal tissue, IRB# 2018–1509; postpubertal tissue, IRB# 2017–1149 and IRB# 2020–3206), following the Declaration of Helsinki. Families and participants provided written informed consent for collection of research specimens. When possible, all minor participants provided assent for the use of research tissue. The collection of human ovarian tissue from deceased organ donors is considered exempt (IRB# 2021–4509) and all clinical information is deidentified. For the purposes of these analyses, specimens from Tanner 1 participants were considered “prepubertal” and those from Tanner 3 to 5 were considered “postpubertal” specimens. See Supplemental Table S1 for participant and donor information.

### Ovarian Tissue Cryopreservation and Oocyte Collection

Samples were collected from participants undergoing OTC for fertility preservation at Ann & Robert H. Lurie Children’s Hospital (Lurie Children’s) from 2018 to 2025. Determination of puberty status and Tanner stage of participants was performed at the time of consent or within 6 months of unilateral oophorectomy for OTC. Ovaries were removed laparoscopically and transferred to the Northwestern Medicine Andrology lab (before 12/2020) or Lurie Children’s Gonadal Tissue Processing Suite (12/2020 onward) for ovarian tissue processing and cryopreservation performed by gonadal tissue processing specialists (32, 33). Briefly, the surgically removed ovary was immediately processed into cortical strips in Oncofertility Consortium (OFC) Holding Media (Sage/Origio no. ART-8040, CooperSurgical).

For isolation of oocytes and cumulus oocyte complexes (COCs) for scPiMS, Holding Media containing tissue fragments that were released as part of the thinning of the ovarian tissue during OTC was transported to the research laboratory where it was passed through a 70 μm strainer (#229484, CELLTREAT). Tissue fragments and cells collected in the strainer were rinsed in L-15 media (#11415114, Life Technologies Corporation) in a 10 cm Petri dish. Denuded oocytes with an intact germinal vesicle and plasma membrane and COCs with ≥3 layers of cumulus cells surrounding an oocyte were identified under a microscope (Olympus SZX10) and then transferred to a charged glass slide (#1358W, Globe Scientific) in minimal media (<10 μl) using a Stripper pipettor and 125 or 275 μm tips (#MXL3-STR, ORIGIO). In some cases, denuded oocytes were obtained from COCs through incubation with 0.3% hyaluronidase solution (#0210074090, MP Biomedicals) in L-15 for 15 to 30 min at 37 °C followed by mechanical disruption using a stripper pipettor. Immediately after the COC or oocyte was deposited onto the slide with the stripper pipettor, small droplets (∼5–10 μl) of 70%, 80%, and 90% ethanol were added sequentially to lightly fix the cells onto the slide. Each droplet was administered immediately after evaporation of the previous solution. Slides were then transferred to −80 °C and stored until data collection.

For isolation of oocytes and follicles for top-down library generation, tissue pieces previously preserved in Cryostor (#07959, STEMCELL Technologies) were thawed in a 37 °C water bath, washed once in Dulbecco's modified Eagle's medium (DMEM)/F12 (#DFL13-6X50 Ml, Caisson Labs), and enzymatically digested as previously described (34). Briefly, tissue fragments were incubated in media containing 40 μg/ml Liberase DH (#05401089001, Sigma-Aldrich), 1000 U DNAse I (#10104159001, Sigma-Aldrich), and 0.4 mg/ml Collagenase IV (#C5138–100 mg, Sigma-Aldrich) in alphaMEM + Glutamax (#32–561–037, Thermo Fisher Scientific) supplemented with 1X insulin-transferrin-selenium (#25–800-CR, Corning), 1X antibiotic-antimycotic (#ABL02–100 Ml, Caisson Labs), and 1 mg/ml human serum albumin (#A9511-5G, Sigma-Aldrich). Digestion proceeded for a maximum of 30 min at 37 °C, 5% CO_2_, shaking at 120 rpm and was quenched by addition of DMEM/F12 supplemented with 10% fetal bovine serum (#F2379-5G, Sigma-Aldrich). The digest was passed through a 70 μm strainer into PBS, then oocytes and follicles were immediately isolated and washed 3x in PBS. PCR tubes containing oocytes and follicles were flash frozen in liquid nitrogen, then all digestion media/PBS was pooled into a microcentrifuge tube and spun at 1500 rpm for 5 min. Media/PBS was removed and pelleted oocytes and follicles were flash frozen. All tubes were stored at −80 °C until data collection.

### SNP Validation

DNA was extracted from cryopreserved cells or tissue for the donors indicated using the Quick-DNA Miniprep Plus Kit (#D4068T, Zymo Research) according to manufacturer’s instructions. Single-nucleotide polymorphism (SNP) genotyping assays for rs496530 (custom assay AN49H7J redesigned from #C__1065759_10, Thermo Fisher Scientific) and rs561930 (#C__3249981_1_, Thermo Fisher Scientific) were conducted in duplicate according to manufacturer’s instructions using TaqMan Genotyping Master Mix (#4371353, Thermo Fisher Scientific) and the QuantStudio 6 Flex System (Thermo Fisher Scientific).

### Microscopy of Single Oocytes and COCs

Low-resolution bright-field microscopy images were taken of all glass slides with fixed, denuded oocytes prior to scPiMS analysis with a PathScan Enabler (Meyer Instruments). High resolution bright-field images of COCs were taken after thawing, after ethanol wash, and after scPiMS analysis on a BioTek Lionheart FX Automated Microscope (Agilent) at 4x and 10x magnifications. Dark-field images of the scPiMS liquid probe and COCs were taken using a Dino-Lite Edge Plus (Dino-Lite) camera at ∼700x magnification.

### Top-Down Mass Spectrometry Library Generation

For liquid chromatography tandem mass spectrometry analysis, the separation was performed on an UltiMate 3000 LC system (Thermo Fisher Scientific) connected to the Orbitrap Exploris 480 mass spectrometer (Thermo Fisher Scientific). Prior to separation, human follicles (oocytes and granulosa cells) were pooled and directly suspended in 20 μl of the liquid chromatography tandem mass spectrometry mobile phase A (0.2% v/v formic acid, 5% v/v acetonitrile [ACN]) and centrifuged at 14,000 g for 30 min. The supernatant was collected and 10 μl was injected into a reversed-phase liquid chromatography capillary column that was prepared in-house (75 μm I.D., 25 cm length, Agilent PLRP-S beads, 5 μm particle size, 1000 Å pore size). The reversed-phase liquid chromatography separation gradient was maintained at 5% mobile phase B (0.2% v/v formic acid, 95% v/v ACN) from 0 to 10 min, and then ramped from 5% B to 15% B over 2 min, from 15% B to 35% B in 58 min, from 35% B to 95% B in 5 min, and then maintained at 95% B for 15 min at a flow rate of 500 nl/min. The mass spectrometer source settings were set as follows: source temperature, 320 °C; RF lens, 200%; ionization voltage, 2000 V. Settings for the full MS1 scan were as follows: resolution, 120,000 (at *m/z* 200); *m/z* range, 500 to 2500; automatic gain control (AGC) target, 200%; maximum injection time, “Auto”. The data were collected in data-dependent acquisition mode using a ‘Top N’ loop count where the N most abundant proteoforms detected in each MS1 scan were selected for MS/MS fragmentation. Fragmentation was performed with the following settings: isolation window, 2*m/z*; higher-energy collisional dissociation collision energy, 32%; resolution, 60,000 (at *m/z* 200); *m/z* range, 300 to 2000, AGC target, 1000%; maximum injection time, “Auto”; ion intensity threshold, 5e3; dynamic exclusion window, 60 s. Results were searched against the SwissProt human proteome database using TDPortal and were exported to TDViewer (Proteinaceous).

### scPiMS Data Collection

Denuded oocytes fixed on a glass slide were thawed under desiccation for ∼5 min to remove excess moisture and submerged in 90% and 100% ethanol solutions for 30 s each to remove excess buffer and enhance protein extraction before desiccation for a final ∼5 min. This process was repeated for COCs.

Denuded oocytes were rastered over with a ∼100 μm dynamic liquid bridge (60% ACN, 5% acetic acid vol/vol) flowing solvent at 500 nl/min and moving at 5 μm/s. The liquid bridge utilized two sampling probes: the primary and the secondary capillary (150 μM OD; 40 μM ID, Polymicro). The nano-DESI sampling probes were assembled as previously described (35, 36). The primary capillary was heated over an open flame and manually extruded to produce a finely pulled tip which was clipped under a microscope to produce an opening for solvent flow. The secondary capillary was not extruded and was positioned close to the MS inlet. A liquid bridge was maintained between the two capillaries to allow direct sampling of the cells and continuous delivery of extracted analytes to the MS. The strip step between the line scans was set to 100 μm to ensure total coverage of the oocyte and promote extraction across the entire cell. Extraction solvent was flowed at 500 nl/min to rapidly extract and dilute proteins for I^2^MS analysis on a QExactive HF Mass Spectrometer (Thermo Fisher Scientific) modified with a 1 kV center electrode voltage. Each oocyte within a COC was sampled by parking the liquid bridge directly on the oocyte to exclude surrounding cumulus granulosa cells, and ions were collected for ∼60 min to maximize ion extraction. Cumulus granulosa cells in each COC were sampled by moving the liquid bridge slowly over the entire cumulus granulosa cell population from a single COC, excluding the oocytes, for ∼30 min.

I^2^MS data were collected with the following settings: Resolution, 70,000 (∼1 s/scan); ionization voltage, 2.8 kV; in-source CID, 15 eV; S-lens radio frequency level, 60%; ion transfer tube temperature, 325; trapping gas, 0.2 (ultrahigh vacuum pressure <2 x 10^-11^ torr); scan range of 500 to 2500*m/z*; enhanced Fourier transform, off; averaging, 0; microscans, 1; AGC, fixed injection time. Injection time was set to 5 ms for denuded oocytes and COCs unless otherwise noted.

### Data Analysis

Ions collected from COCs and denuded oocytes were processed to produce true mass domain spectra as previously described (27) using the commercially available processing software STORIboard (Proteinaceous). Signal-to-noise set to 0, R^2^ = 0.98, duration threshold set to 0.42, a maximum time of birth of 0.2, a bin size of 5 ppm, number of charge neighbors set to 2, number of isotope neighbors set to 5, and minimum ions in bin set to 1. Data from cumulus granulosa cells were processed identically.

I^2^MS data processed in STORIBoard were subjected to intact mass tag searching as previously described (27). Spectra were calibrated using abundant proteoforms that were identified repeatedly in most oocytes and COC samples (Supplemental Table S2). Briefly, a custom version of TDValidator (Proteinaceous) implemented with an MS^1^ intact mass tag search function was used to search oocyte and cumulus cell data against databases created from bottom-up proteomics results from single oocytes (17, 18, 19) (Supplemental Table S3) or ovarian tissue (29, 37) (Supplemental Table S4), respectively. Candidate proteoforms were generated *in silico* with or without an initiator methionine and N-terminal acetylation. Data were searched using a mass tolerance of 5 ppm and a minimum score threshold of 0.3 to assign proteoform features. The top-down library (Supplemental Table S5) was used to supplement the bottom-up database search results. Additional proteoform identifications, including phosphorylations and SNP, were made based on manual inspection and existing UniProt entry information. All matching proteoforms were manually validated in TDValidator to remove false positives and duplicate proteoforms.

Proteoform features in the spectra were detected using THRASH deconvolution in TDValidator. The following settings were used for all spectra: S/N Cutoff, 1; Maximum mass, 70,000 Da; Maximum charge state, 1; Minimum RL value, 0.975.

### Experimental Design and Statistical Rationale

Denuded oocytes collected from six different donors and COCs collected from two donors were analyzed via scPiMS. Only participants with two or more collected oocytes were included in this study to gauge reproducibility between analyzed oocytes of the same tissue origin. Only proteoforms that were identified in 50% or more (>12) of the denuded oocytes were utilized for interdonor comparisons and analysis of age-related changes (Supplemental Fig. S1).

Gene Ontology (GO) analysis was performed using PANTHER (https://geneontology.org/) (38, 39, 40). Specifically, a list of UniProt accessions for all 28 oocytes and cumulus cell samples were submitted to PANTHER for GO analysis (Supplemental Tables S6 and S7). The results contain the top-level GO cellular components or protein classes associated with the submitted UniProt accessions with false discovery rate-corrected *p* values.

(1) $$\text{Similarity}=\frac{\left(\#\;\text{of}\;\text{Overlapping}\;\text{IDs}\right)*2}{\#\;\text{of}\;\text{IDs}\;\text{in}\;\text{Oo}1+\#\;\text{of}\;\text{IDs}\;\text{in}\;\text{Oo}2}$$

Oocyte clustering was performed by scoring the overlap in protein identifications between two oocytes via Equation 1. Oocytes that scored highly when compared were clustered closer together. These results were used to create dendorgrams and heatmaps to gauge oocyte similarity.

**Fig. 1:**
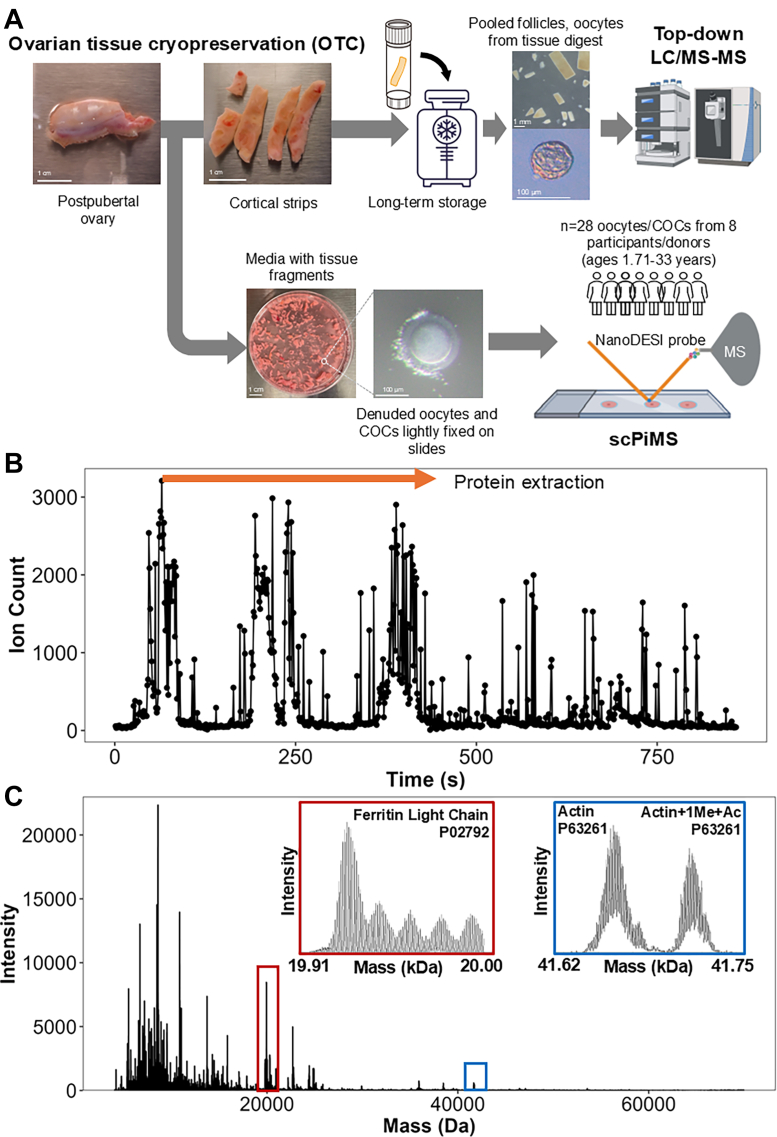
**Workflow summary for the scPiMS analysis of human oocytes isolated during OTC**. *A*, overview of the OTC process during which oocytes and COCs are released from the ovarian tissue into the processing media and are then isolated and fixed on glass slides for scPiMS analysis. *B*, exemplary total ion chronogram for an oocyte analyzed via scPiMS; ion count peaks represent the time when the scPiMS probe contacts the oocyte and extracts proteins. *C*, exemplary mass spectrum for a single oocyte with insets highlighting isotopically resolved peaks corresponding to ferritin light chain (*red*) and actin (*blue*). COC, cumulus oocyte complex; OTC, ovarian tissue cryopreservation; scPiMS, single-cell proteoform imaging mass spectrometry.

**Fig. 2:**
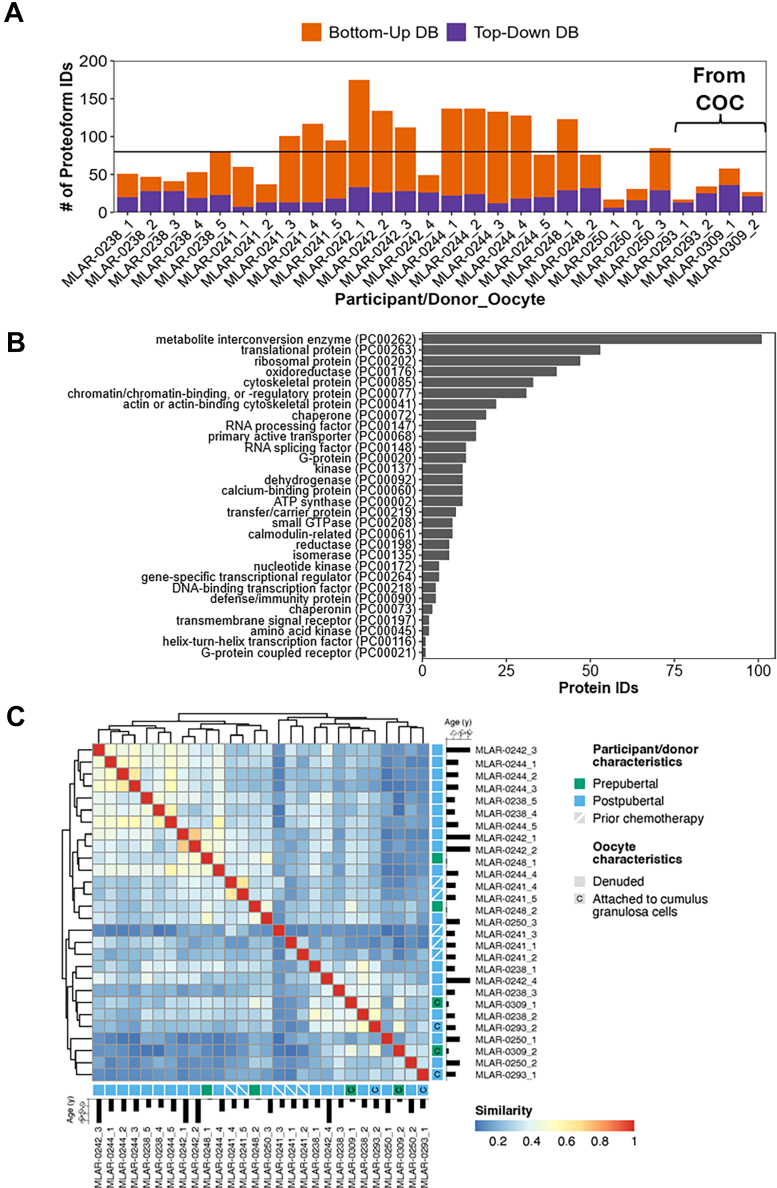
**Summary of the results from the scPiMS analysis of 24 denuded oocytes and four oocytes within a COC**. *A*, the number of proteoform identifications made for each individual oocyte and whether they were identified by the bottom-up library (*orange*) or the top-down library (*purple*). *B*, proteins found in the 28 scPiMS analyzed oocytes categorized into protein classes (135 unclassified proteins are not shown). *C*, similarity heatmap clustering the 28 oocytes by the similarity score calculated in Equation 1, labeled by pubertal status, age, prior chemotherapy, and presence of cumulus cells. scPiMS, single-cell proteoform imaging mass spectrometry; COC, cumulus oocyte complex.

## Results

### scPiMS Analysis of Single Oocytes Isolated During OTC

Single oocytes were isolated from ovarian tissue processing media and denuded to remove cumulus granulosa cells (Supplemental Fig. S2) prior to ethanol fixation on glass slides for scPiMS analysis (Fig. 1*A*). Slides were stored at −80 °C until analysis by scPiMS, at which point they were further washed in increasing concentrations of ethanol to remove any crystallized buffer components from minimal media, which can form during desiccation of the slide, without perturbing the integrity of the cell (Supplemental Fig. S3). Once prepared, single oocytes were subjected to scPiMS and proteoform ions from each oocyte were collected as the sampling probe rastered over the oocyte (Supplemental Fig. S4), causing a large spike in number of detected ions for each scan where the probe made contact (Fig. 1*B*). These ions were then assigned a charge and mass to create a high resolution, true mass spectrum with proteoform features ranging from 4 to 70 kDa (Fig. 1*C*). Within the mass spectra generated for the oocytes, we observed an average of 169 ± 111 proteoform features (maximum of 462, minimum of 22) from a single oocyte. Our average proteoform feature identification rate was ∼55% across all oocytes samples (Supplemental Fig. S5). We found that the manually validated proteoform identifications tended to span larger mass ranges than those detected algorithmically, likely because the algorithm used for feature detection struggles to detect larger proteoforms that are not fully isotopically resolved, but can otherwise be assigned manually based on mass accuracy (Supplemental Fig. S6) (41). The large variation in detected proteoforms could result from small variations in oocyte size and the number of charge-assigned ions extracted from each oocyte.

Oocytes (∼100 μm) yield >10x more ions during scPiMS analysis (Supplemental Fig. S7) than typical somatic cells (∼20 μm) due to the increased protein content in oocytes, allowing for direct proteoform identification without aggregating single cell spectra together (23, 24, 42). Beyond variations in oocyte size, variations in the number of usable ions and proteoform features were also impacted by the rupturing of some oocytes upon adherence to the glass slide (Supplemental Fig. S8). However, the scPiMS probe can still sample protein remaining at the site of the rupture, allowing for identification of oocyte-specific proteins even from the oocyte with the lowest number of charge-assigned ions, MLAR-0250_01.

The direct sampling and high sensitivity afforded by the scPiMS approach enabled us to identify 78 proteoforms on average from a single oocyte, with the highest number exceeding 170 proteoform IDs (Fig. 2*A* and Supplemental Table S6). From the 24 denuded oocytes and the four oocytes probed within a COC, we identified 559 unique proteins and their associated proteoforms (Supplemental Table S7). These proteins correlate strongly with the most abundant proteins identified by bottom-up proteomics on single human oocytes (17, 18, 19). We classified this list of unique proteins into GO protein classes to understand the types of proteins that were found commonly in oocytes using scPiMS (Fig. 2*B*). Besides the proteins that remained unclassified by this approach (135 proteins of 559 total), we found that metabolite interconversion enzymes, translational and ribosomal proteins, oxidoreductases, and cytoskeletal proteins were the most observed classes in oocytes. Proteins implicated as RNA processing factors, protein chaperones, and mitochondrial proteins were also identified in high abundance across the cohort. We further broke down this list of proteins by individual oocytes and performed a pairwise comparison of each oocyte to gauge the similarity of each cell, as calculated by Equation 1 (Fig. 2*C*). From this analysis, we found that while some oocytes from the same participant clustered close together, there was a large amount of heterogeneity in protein identifications that led to low overlap between oocytes of the same participant. Heterogeneity between oocytes from the same participant and general comparisons between the participants are further discussed in the following sections.

### Proteomic Comparisons of Denuded Oocytes and COCs

Oocytes are often isolated with granulosa cells attached (within a COC), necessitating denuding of the cumulus granulosa cells prior to traditional single oocyte proteomic approaches to prevent contamination (17, 18, 19). The high spatial resolution afforded by the scPiMS probe motivated our analysis of oocytes and cumulus granulosa cells within the same COC to identify proteoforms that are specific to either cell type. We began by washing the COCs fixed on glass slides with increasing concentrations of ethanol in an identical fashion to the denuded oocytes. The scPiMS probe was landed on the slide and moved onto the oocyte within the COC while avoiding the surrounding cells (Supplemental Fig. S9) and sampled for 1 h to produce a mass spectrum for the oocyte. When compared to the denuded oocytes that were sampled by rastering with the scPiMS probe, the oocytes sampled directly from COCs had ∼46% fewer identified proteoforms on average and a reduced mass range of 5 to 25 kDa over which identifications were made (Fig. 2*A* and Supplemental Fig. S6). Cumulus granulosa cells (∼8 μm diameter, 10s-100s of cells per COC) were sampled in a similar fashion to the oocytes by slowly moving the probe manually over the cells and collecting proteoform ions for ∼30 min for each COC with cumulus granulosa cells (43). Only three of the COCs used in this study had significant populations of cumulus granulosa cells for sampling (Supplemental Fig. S10). For all COCs analyzed, the number of proteoforms detected from the cumulus granulosa cell population was higher than for the attached oocytes (Supplemental Fig. S11).

**Fig. 3:**
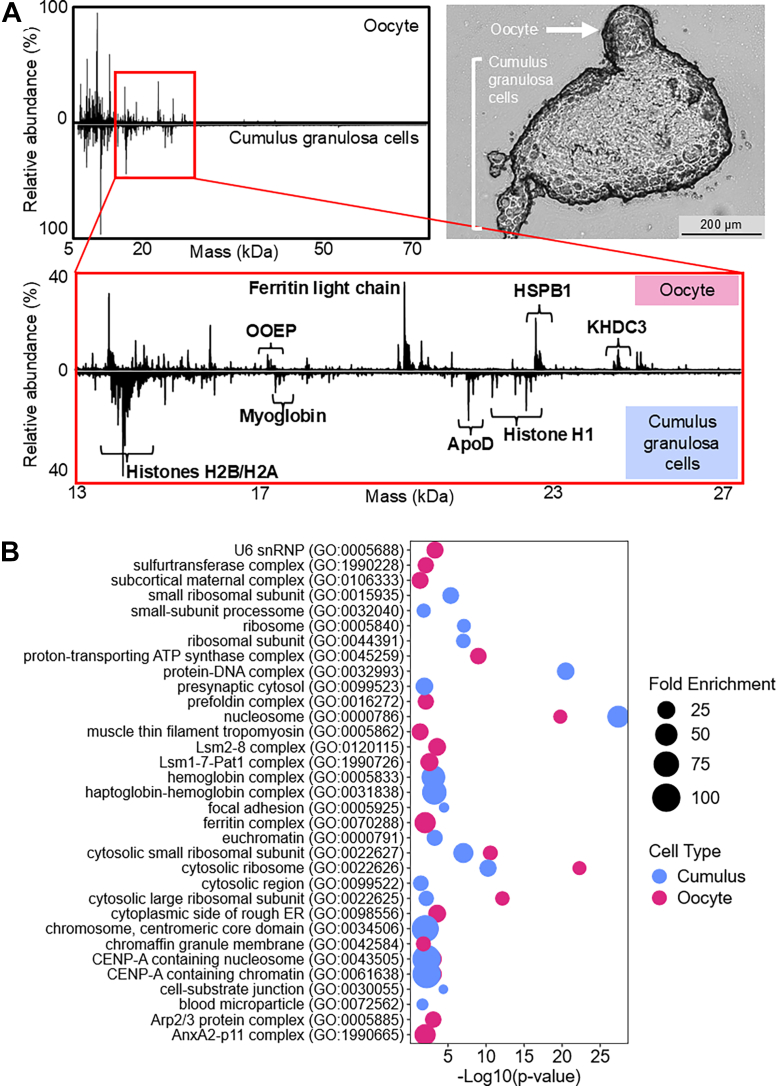
**Comparison of the oocyte and cumulus granulosa cell proteomes**. *A*, bright-field microscopy image of a COC and a butterfly plot comparing exemplary oocyte (*top*) and cumulus granulosa cell (*bottom*) scPiMS spectra with labels identifying proteins specific to oocytes and cumulus granulosa cells. *B*, GO enrichment of cellular components for all detected proteins from oocytes (*red*) and all detected proteins from cumulus granulosa cells (*blue*). scPiMS, single-cell proteoform imaging mass spectrometry; COC, cumulus oocyte complex; GO, Gene Ontology.

To overcome the limitations of scPiMS analysis of oocytes within COCs, exemplary data from denuded oocytes were used to determine protein specificity to oocytes or cumulus granulosa cells. Proteoforms corresponding to oocyte-expressed protein homolog (OOEP, A6NGQ2), ferritin light chain (P02792), heat-shock protein beta 1 (HSPB1, P04792), and KH domain-containing protein 3 (KHDC3, Q587J8) were all highly specific to oocytes, whereas a variety of histones, myoglobin (P02144), and apolipoprotein D (ApoD, P05090) were highly specific to the proteome of cumulus granulosa cells (Fig. 3*A* and Supplemental Table S8). OOEP and KHDC3 are members of the SCMC, an oocyte-specific protein complex that has been implicated in oocyte maturation and embryo development (44). Ferritin light chain and HSPB1 are more ubiquitous proteins that are known to maintain iron homeostasis and cellular oxidative stress and heat shock responses, respectively, indicating these proteins may play a similar role to maintain oocyte health (45, 46).

We sought to expand this cell-type specificity analysis to include GO cellular component enrichment analysis for all unique proteins identified across the cohort of oocytes and cumulus granulosa cells (Fig. 3*B*). Proteins corresponding to cellular components of focal adhesion, cell-substrate junctions, blood microparticles, and other blood-related components like the haptoglobin–hemoglobin complex were only enriched in cumulus granulosa cells (Fig. 3*B*). In contrast, proteins corresponding to the ferritin complex, the SCMC, actin assembly, protein chaperones, and RNA processing were specifically enriched in oocytes (Fig. 3*B*). Proteins related to the ribosome and chromatin were strongly enriched in the cumulus cells compared to the oocytes, suggesting that the needs for protein production and chromatin maintenance are higher for rapidly dividing cells than they are for a cell that was previously arrested in prophase I of meiosis I (Fig. 3*B*). These results are consistent with previously published results which found that the oocyte proteome is more specialized toward homeostasis while somatic cells are geared toward chromatin organization, translation, and transcription (17).

### Intraparticipant and Interparticipant Oocyte Proteome Heterogeneity

**Fig. 4:**
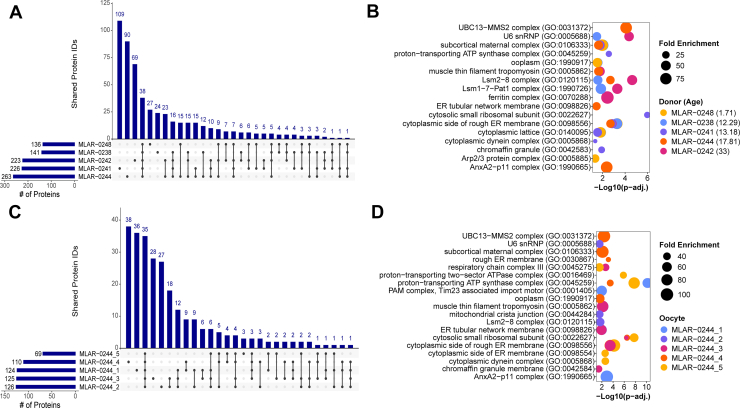
**Intradonor and interdonor oocyte proteome heterogeneity**. *A*–*B*, comparison of the overlapping oocyte protein identifications (*A*) and top enriched GO cellular component features (*B*) from five individual donors. *C*–*D*, comparison of overlapping oocyte protein identifications (*C*) and top enriched GO cellular component features (*D*) for five oocytes from the same donor. GO, Gene Ontology.

Following our result from the similarity clustering (Fig. 2*C*), we wanted to compare proteomes of oocytes from the same individual versus among multiple donors of different ages. For evaluating interparticipant similarity, we combined the protein identifications from each oocyte for 5 of the 6 donors whose oocytes were denuded prior to scPiMS analysis and calculated the overlap in protein identifications (Fig. 4*A* and Supplemental Fig. S12*A*). Oocytes from MLAR-0250 (age 19.93 years) were excluded from this analysis due to the high variability in number of proteoform identifications per oocyte (Fig. 2*A*). There were 38 (7%) protein IDs that overlapped between oocytes from the remaining five donors. Notably, oocytes from the two oldest donors in this comparison, MLAR-0244 (17.81 years) and MLAR-0242 (33 years), had the highest degree of exclusive overlap with 23 protein IDs found exclusively in oocytes from these two donors (Fig. 4*A*). Oocytes from donors MLAR-0241 (13.18 years), MLAR-0244 (17.81 years), and MLAR-0242 (33 years) had the greatest number of unique protein identifications with 109 (20%), 90 (17%), and 69 (13%) of the protein IDs found exclusively in oocytes from these three donors (Fig. 4*A*).

We next performed GO enrichment analysis to identify key cellular components represented by proteins that were enriched in specific participants (Fig. 4*B*). We found that oocytes from all participants had a similar enrichment for SCMC, cytoplasmic lattice, and the cytoplasmic side of rough endoplasmic reticulum (ER) membrane pathways (Fig. 4*B*). The enrichment of the SCMC in all the participants is indicative of the role of this complex in coordinating key developmental processes in oocytes like meiotic spindle formation, genome-wide DNA methylation, and DNA repair (44, 47). The ER plays important roles in regulating calcium signaling and the removal of misfolded proteins, and the cytoplasmic side of the ER specifically serves as a site for synthesis, folding, and posttranslational modification for secreted proteins (48), all processes that play key roles in oocyte development and function (17, 47, 49). Beyond similarities among the participants, we also identified several components that were highly enriched in oocytes from specific participants. We found that components related to the LSM1-7-Pat1 (p-adj=4.94E-4) and LSM2-8 (p-adj. = 2.34E-5) complexes were highly enriched in the oldest donor, along with U6 snRNP (p-adj. = 4.41E-5) and ferritin complex (p-adj. = 3.73E-3) (Fig. 4*B*). We identified a variety of proteins associated with the LSM1-7-Pat1 and LSM2-8 complexes, including LSM1, 3, 4, 6, and 7. The only donor whose oocytes contained all of these LSM proteins was MLAR-0242 (33 years old) (Supplemental Fig. S13). The LSM1-7-Pat1 and LSM2-8 complexes are required for mRNA decay (50) and the U6 snRNP is required for RNA splicing (51), suggesting a potential importance of mRNA maintenance in oocytes as age increases (52). When we looked at corresponding cellular components that were overrepresented by proteins in our dataset, in participant MLAR-0241 (13.18 years), components related to chromaffin granules (protein secretion, p-adj. = 1.23E-2), mitochondrial proteins (p-adj. = 2.8E-3), and cytoplasmic dynein complex (p-adj. = 4.56E-2) were specifically enriched (Fig. 4*B*). Though protein secretion and mitochondrial activity are generally important for intercellular communication and energy generation, the cytoplasmic dynein complex has been implicated in the reorganization of the ER during oocyte maturation in mice (53).

Besides analyzing the proteomic heterogeneity of oocytes from different participants, we also sought to understand the heterogeneity of the oocyte population from a single participant. For this assessment, we selected participant MLAR-0244 due to the low variability in the number of proteoform identifications for each oocyte (Fig. 2*A*). Thirteen percent (35 of 263 total) of protein IDs from MLAR-0244 were shared among the oocytes, which is double the number of protein IDs (38 of 540 total: 7%) that were shared among oocytes from all participants (Fig. 4, *A* and *C*, and Supplemental Fig. S12*B*). For the four MLAR-0244 oocytes with similar numbers of proteoform identifications, unique protein IDs per oocyte ranged from 18 (7%) to 38 (14%), while MLAR-0244_5 only had 3 (1%) unique protein IDs, likely due to the reduced number of identifications made for that oocyte (Fig. 4*C*). When we analyzed proteins identified in MLAR-0244 by GO cellular component enrichment, we found some components conserved between oocytes, like the SCMC and cytoplasmic lattice, as well as some that were only enriched in a specific oocyte (Fig. 4*D*). For example, proteins in oocyte MLAR-0244_2 corresponded to mitochondrial function components (mitochondrial contact site and cristae organizing system [MICOS] complex and the mitochondrial crista junction, p-adj. = 2.56E-2) and RNA processing (U6 snRNP (p-adj. = 2.59E-2) and LSM2-8 complex (p-adj. = 2.00E-2)) (Fig. 4*D*). We also observed the enrichment of secretory machinery (chromaffin granule membrane, p-adj. = 3.71E-2), cytosolic ribosome (p-adj. = 4.08E-7), ER tubular network membrane (p-adj. = 1.17E-2), and muscle thin filament tropomyosin (p-adj. = 7.14E-3) exclusively in proteins from oocyte MLAR-0244_3 (Fig. 4*D*). The unique cellular components corresponding to individual oocytes demonstrate the utility of scPiMS to capture the heterogeneity in oocyte proteomes within individual oocytes from a single participant; these results may be indicative of the differently sized antral follicles from which these oocytes and COCs came and or may reflect developmental competence and progression towards dominance for ovulation.

### Donor-specific Proteoform Landscapes of Subcortical Maternal Complex Members

**Fig. 5:**
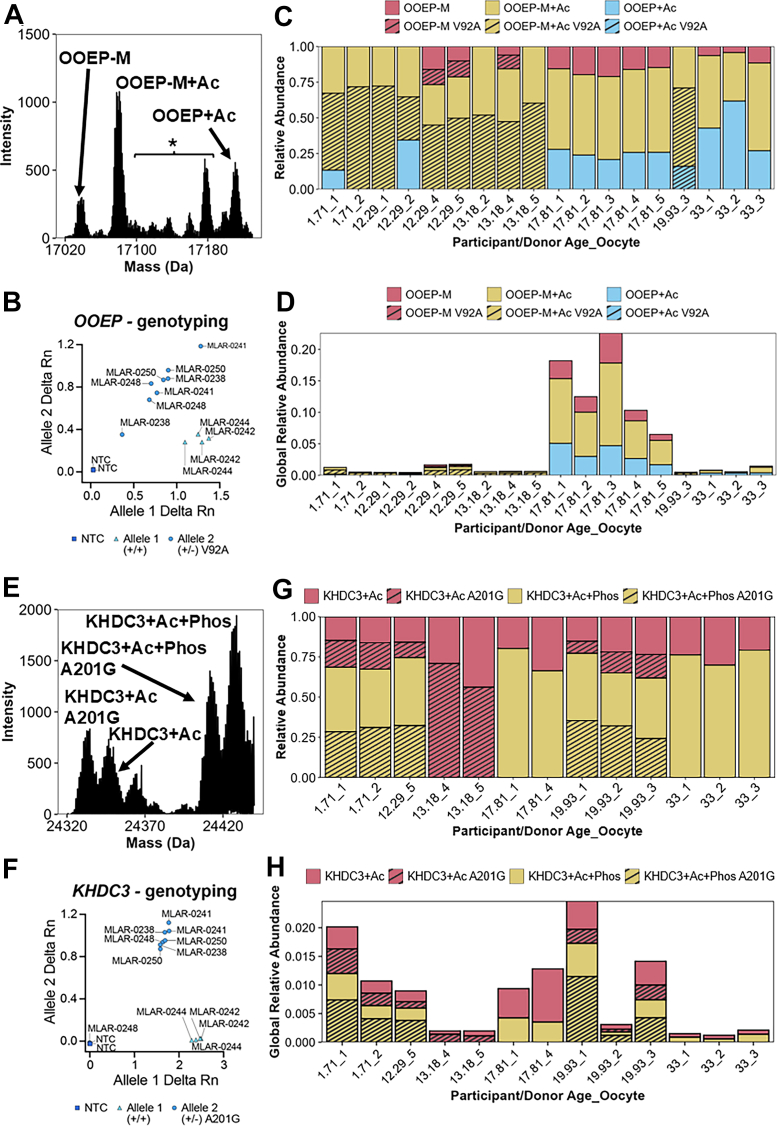
**Changes in the proteoform landscape for subcortical maternal complex members OOEP and KHDC3**. *A*–*D*, exemplary mass spectrum of OOEP proteoforms (*A*), qPCR genotype validation of identified *OOEP* (+/−) V92A SNP variants (*B*), relative (*C*) and global abundance (*D*) of OOEP proteoforms in all oocytes in which OOEP was detected. *E*–*H*, exemplary mass spectrum of KHDC3 proteoforms (*E*), qPCR genotype validation of identified *KHDC3* (+/−) A201G SNP variants (*F*), relative (*G*) and global (*H*) abundance of KHDC3 proteoforms in all oocytes in which KHDC3 was detected. Example spectra from each participant are shown in Supplemental Fig. S11. ∗ = Unannotated, NTC, no template control. OOEP, oocyte-expressed protein homolog; qPCR, quantitative PCR; SNP, single-nucleotide polymorphism.

One advantage offered by scPiMS compared to bottom-up methods for protein detection is the unambiguous detection of intact proteoforms directly from single cells. Utilizing scPiMS, we detected two oocyte-specific proteins, OOEP and KHDC3, and their related proteoforms in more than half of all the denuded oocytes that were analyzed (Fig. 5, *A* and *E*, and Supplemental Fig. S14). In addition to the canonical proteoforms for OOEP and KHDC3, we also identified peaks corresponding to SNPs (Fig. 5, *A* and *E*, and Supplemental Fig. S14). We identified a peak corresponding to a V92A substitution in OOEP in more than half of the included donors (Supplemental Fig. S14). We also identified a peak corresponding to a A201G substitution in KHDC3 in the same four donors carrying the SNP for OOEP. The frequency of these SNPs in our dataset was 66% (4/6 donors) which is close to the ∼50% frequency found in a global population study performed previously (54). We validated these SNP annotations by utilizing genomic DNA extracted from donor samples (ovarian tissue or peripheral blood mononuclear cells) and found our assignments to be accurate (Fig. 5, *B* and *F*).

In addition to SNP variants of OOEP and KHDC3, we also identified a variety of other proteoforms and considered the relative abundance of these proteoforms across the entire cohort (Fig. 5, *C* and *G*). We identified three major OOEP proteoforms with or without the V92A SNP: OOEP without the initiator methionine (OOEP-M), OOEP-M with an acetylation (OOEP-M + Ac), and OOEP with an acetylation (OOEP + Ac) (Fig. 5*C* and Supplemental Table S9). We found two KHDC3 proteoforms of both the major allele and A201G SNP: KHDC3 with an acetylation (KHDC3+Ac) and KHDC3 with an acetylation and a phosphorylation (KHDC3+Ac + Phos) (Fig. 5*G* and Supplemental Table S9). To the best of our knowledge, no previous works have identified any posttranslational modification on OOEP, so the significance of the variable proteoform landscape associated with this protein is unclear. On the other hand, the phosphorylation on KHDC3 is required for its function in mediating DNA double-stranded break repair and maintaining genomic stability (47). Though both OOEP and KHDC3 have been previously identified in single human oocyte proteomic studies (17, 18, 19), their associated PTMs and distinct proteoforms have not been previously identified.

Having identified these proteoforms in multiple oocytes from donors across the cohort age range, we sought to understand how these proteoforms varied in abundance with age. Since total protein abundance was variable among oocytes, we considered the relative abundances of OOEP and KHDC3 proteoforms for each oocyte in which they were identified (Fig. 5, *C* and *G*). For the OOEP proteoforms in oocytes from younger donors (<17 years), we generally found that the OOEP-M + Ac and its SNP counterpart were the dominant proteoforms, with much smaller contributions from OOEP + Ac and the major allele and SNP forms of OOEP-M (Fig. 5*C*). However, in oocytes from older postpubertal donors (>17 years), the OOEP proteoform landscape included more abundant OOEP + Ac and OOEP-M, with the highest proportion of OOEP + Ac observed in the oldest (33 years) donor (Fig. 5*C*). In contrast, KHDC3 proteoforms were remarkably consistent in oocytes from donors across the entire age range (Fig. 5*G*), with ∼75% of total KHDC3 phosphorylated regardless of SNP presence. This remarkable consistency is likely indicative of the role that phosphorylation plays for KHDC3 function (47). However, in oocytes from MLAR-0241 (13.18 years), we found that KHDC3 completely lacked phosphorylation and only contained KHDC3+Ac proteoforms (Fig. 5*G*).

In addition to exploring relative abundance of different proteoforms, we also considered how the global abundance of KHDC3 and OOEP differed with age. We found that the global relative abundance of OOEP was highest across oocytes from MLAR-0244 (17.81 years old) and was relatively consistent at much lower levels throughout the rest of the age range. (Fig. 5*D*). KHDC3 was most abundant in oocytes from the two youngest donors, MLAR-0248 (1.71 years old) and MLAR-0238 (12.29 years old), and again in oocytes from MLAR-0244 and MLAR-0250 (19.93 years old) (Fig. 5*H*). In a previous study, KHDC3 and OOEP mRNA transcripts were measured in oocytes isolated from different size antral follicles from prepubertal, adult, and advanced age sheep ovaries. KHDC3 and OOEP mRNA transcripts were differentially expressed among different oocyte diameters and across ages. Additional studies in human oocytes are required to fully understand the dynamic production of these two proteins during oogenesis and across the lifespan (55). Overall, scPiMS analysis of single oocytes allowed proteoform level insights of OOEP and KHDC3 and identified donor-specific proteoform features that would otherwise be missed by bottom-up or affinity-reagent methodologies, cementing scPiMS as a promising new platform for single-cell analysis.

## Discussion

Single-cell analysis of proteins is a powerful approach for understanding biological processes from limited and intrinsically heterogeneous cell populations. One example of this is the recent interest in applying single cell proteomics to oocytes collected for the purpose of assisted reproductive technologies (ART) to improve the outcomes of IVF and better understand what constitutes high quality oocytes that can be matured into eggs, that once fertilized, develop into embryos. Although ovarian stimulation and egg retrieval for IVF is the main fertility preservation option available to most women, many are poor responders to ovarian stimulation, have diagnoses incompatible with the procedure, or simply lack access to ART services (56, 57), (https://www.hhs.gov/about/news/2024/03/13/fact-sheet-in-vitro-fertilization-ivf-use-across-united-states.html). In addition, prepubertal patients undergoing gonadotoxic treatments do not yet produce mature eggs and thus cannot utilize IVF. Therefore, improving IVM of oocytes holds great potential to provide millions of women with access to fertility preservation. The low quantity of oocytes available for research precludes analysis of these cells by traditional proteomics approaches and requires new tools for understanding oocytes at the molecular level (58, 59). Though several studies have performed single-cell proteomics measurements on human oocytes, all of them rely on peptide level identification of proteins (17, 18, 19). We leveraged the high sensitivity of scPiMS analysis to move beyond peptide level analysis of oocyte proteomes and measure intact proteoforms in oocytes from girls and women aged 2 to 33 years that underwent OTC. We identified 559 unique proteins and 769 unique proteoforms from 24 denuded oocytes and four oocytes analyzed within COCs from eight participants/donors with an average of 78 unique proteoforms identified per oocyte. We identified that these maturing oocytes are consistently heterogeneous, with relatively low correlation between single oocytes even from the same donor, likely due to variable stages of differentiation of the antral follicles from which these germinal vesicle oocytes were released.

Key advantages of utilizing scPiMS over peptide-based single cell proteomics are the ability to analyze intact oocytes and the high spatial resolution afforded by the probe. Utilizing intact oocytes fixed onto glass slides allows for direct proteoform extraction and detection. In addition, coupling direct extraction with I^2^MS detection efficiently utilizes the mass spectrometer as a separation approach, reducing the potential loss of material due to sample handling and contact with other instrument machinery. The use of I^2^MS also enhances sensitivity by funneling ions of any charge into a single isotopic distribution for a given proteoform, reducing sensitivity loss from signal splitting into multiple charge state distributions (30, 31). The high spatial resolution of the scPiMS probe allows for *in situ* analysis of different cell populations without disrupting the functional tissue unit; here, we collected data from oocytes within COCs as well as the surrounding cumulus granulosa cells, which allowed us to assess proteoform specificity to oocytes. Although we could selectively sample oocytes directly from COCs, the number of proteoforms identified from this approach was generally lower than what was possible when analyzing denuded oocytes. This may be due to many facets of the oocyte being occluded by the dense cumulus granulosa cells, which could reduce the surface area of the oocyte that is accessible by the extraction solvent. Despite these challenges, we were able to identify several oocyte-specific proteoforms that did not appear in cumulus granulosa cell spectra, including OOEP, KHDC3, ferritin light chain, and HSPB1. Beyond those examples, we leveraged full proteomic profiles to identify differential enrichment of cellular components, allowing us to more accurately describe the specialization of the oocyte proteome compared to that of the cumulus granulosa cells.

One key finding in our study was that the proteomes of maturing oocytes isolated during OTC were heterogeneous between participants of different ages and even among oocytes from the same participant. We found that n = 5 oocytes from the same participant exhibited greater similarity to one another than did oocytes from different participants, with 13% overlapping IDs between all five oocytes from the same participant compared to just 7% for oocytes from five participants. Common proteins among oocytes included OOEP, ferritin light chain, ubiquitin, calmodulin, and peptidyl-prolyl cis-trans isomerase B. Oocytes from only the youngest participant (MLAR-0248) lacked secretory proteins identified in all other participants/donors, including Annexin A7, DLRB1, DYL2, and DYL1. This could indicate the reduced secretory activity and the overall reduced quality of maturing oocytes from very young children (60). In the intraparticipant comparison, we found that oocytes MLAR-00244_1 to 3 contained RTN4 and LNP, both key proteins for developing proper ER morphology, whereas oocytes 4 and 5 did not. This pattern was identical for MICOS complex proteins MIC13, and MIC27. LSM4, 6, 7 were also selectively identified in the first three oocytes. This pattern suggests true biological heterogeneity, where some oocytes have multiple proteins related to similar cellular processes and others lack them completely. This is distinct from the inherent technical noise expected when sampling from multiple samples and demonstrates the potential to uncover both conserved and divergent biological processes among maturing oocytes using this technology.

scPiMS analysis of single human oocytes is a promising approach to identify intact proteoforms and understand how proteoform landscapes in single cells change with biological variables. However, one key limitation of this approach is the lack of fragmentation MS^2^ data generated during the analysis. Unlike PiMS analysis of tissue (28), single cells are too small and provide too little protein material to generate top-down fragmentation data of identified proteoforms to confirm proteoform identity and localize posttranslational modifications. We aim to expand our ability to perform MS2 measurements directly from single cells by improving the sensitivity of scPiMS through additional technical and methodological development. The development of additional comprehensive proteomic libraries and atlases of diverse human tissues will also aid with identifications made by database searching. We also aim to further improve the sample processing prior to scPiMS analysis to reduce variation in proteoform identifications that could be driven by ionization suppression arising from chemical contaminants on the glass slides. In addition, we aim to further automate scPiMS analysis to enable rapid, reproducible analysis of single cells at larger scales to enhance the feasibility of answering clinical questions with this approach.

Despite these challenges, we utilized the high mass accuracy afforded by I^2^MS analysis to match theoretical proteoform masses with masses in our datasets, assigning peaks with mass errors lower than 5 ppm, with most identifications made with even smaller mass error (<3 ppm) (Supplemental Fig. S15). This high mass accuracy facilitated the confident identification of OOEP and KHDC3 proteoforms, including proteoforms containing known SNPs and modifications required for function. Some of the most striking results were the variability in N-terminal methionine removal and acetylation of OOEP and the consistent phosphorylation stoichiometry of KHDC3. Although little is known about the specific functional roles of methionine excision and N-terminal acetylation in protein function, they are understood to affect protein stability and influence the degradation of a protein (61, 62). Though phosphorylation of KHDC3 is a known modification associated with this protein’s function (47), we did identify KHDC3 proteoforms in two oocytes from donor MLAR-0241 that completely lacked this phosphorylation. This participant was the only one in the cohort to receive gonadotoxic chemotherapy prior to undergoing OTC, which could contribute to the lack of observed KHDC3 phosphorylation. Additional studies focusing on the structure-function relationship of these altered proteoforms would help illuminate the role these may play in maturing oocytes.

Single oocyte analysis by scPiMS provides a new view of intact proteoforms and diverse proteomes associated with single cells. We identified proteins associated with key oocyte biological processes and were able to distinguish single oocytes from one another on this basis. Using this technique, we were able to observe novel changes to the proteoform landscapes for OOEP and KHDC3 in oocytes from participants and donors of a previously unassessed age range. These initial proteoform-aware observations of single oocytes will serve as a unique resource to understand oocyte proteomes throughout childhood and adolescence and inform continued development of IVM approaches for immature oocytes to meet the needs of more women seeking ART.

## Data Availability

The MS-based proteomics data have been deposited into the MassIVE database under identifier MSV000099842.

## Supplemental Data

This article contains supplemental data.

## Conflict of Interest

N. L. K. and J. O. K are involved in the commercialization of I^2^MS technology with Thermo Fisher Scientific. The rest of the authors declare no competing interests.

## References

[1] Telfer E.E., Grosbois J., Odey Y.L., Rosario R., Anderson R.A. (2023). Making a good egg: human oocyte health, aging, and *in Vitro* development. Physiol. Rev..

[2] Barton S.E., Najita J.S., Ginsburg E.S., Leisenring W.M., Stovall M., Weathers R.E. (2013). Infertility, infertility treatment, and achievement of pregnancy in female survivors of childhood cancer: a report from the childhood cancer survivor study cohort. Lancet Oncol..

[3] Dolmans M.M., Hossay C., Nguyen T.Y.T., Poirot C. (2021). Fertility preservation: how to preserve ovarian function in children, adolescents and adults. J. Clin. Med..

[4] Kim S., Lee Y., Lee S., Kim T. (2018). Ovarian tissue cryopreservation and transplantation in patients with cancer. Obstet. Gynecol. Sci..

[5] Wallace W.H.B., Thomson A.B., Kelsey T.W. (2003). The radiosensitivity of the human oocyte. Hum. Reprod..

[6] Donnez J., Dolmans M.M. (2013). Fertility preservation in women. Nat. Rev. Endocrinol..

[7] Donnez J., Dolmans M.M. (2017). Fertility preservation in women. N. Engl. J. Med..

[8] Wallace W.H.B., Smith A.G., Kelsey T.W., Edgar A.E., Anderson R.A. (2014). Fertility preservation for girls and young women with cancer: population-based validation of criteria for ovarian tissue cryopreservation. Lancet Oncol..

[9] Poirot C., Abirached F., Prades M., Coussieu C., Bernaudin F., Piver P. (2012). Induction of puberty by autograft of cryopreserved ovarian tissue. Lancet.

[10] Demeestere I., Simon P., Dedeken L., Moffa F., Tsépélidis S., Brachet C. (2015). Live birth after autograft of ovarian tissue cryopreserved during childhood. Hum. Reprod..

[11] Donnez J., Dolmans M.M., Demylle D., Jadoul P., Pirard C., Squifflet J. (2004). Livebirth after orthotopic transplantation of cryopreserved ovarian tissue. Lancet.

[12] Dolmans M.M., von Wolff M., Poirot C., Diaz-Garcia C., Cacciottola L., Boissel N. (2021). Transplantation of cryopreserved ovarian tissue in a series of 285 women: a review of five leading European centers. Fertil. Steril..

[13] Dolmans M.M., Luyckx V., Donnez J., Andersen C.Y., Greve T. (2013). Risk of transferring malignant cells with transplanted frozen-thawed ovarian tissue. Fertil. Steril..

[14] Karavani G., Wasserzug-Pash P., Mordechai-Daniel T., Bauman D., Klutstein M., Imbar T. (2021). Age-dependent *in Vitro* maturation efficacy of human oocytes – is there an optimal age?. Front. Cell Dev. Biol..

[15] Gruhn J.R., Zielinska A.P., Shukla V., Blanshard R., Capalbo A., Cimadomo D. (2019). Chromosome errors in human eggs shape natural fertility over reproductive life span. Science.

[16] Karavani G., Schachter-Safrai N., Revel A., Mordechai-Daniel T., Bauman D., Imbar T. (2019). *In Vitro* maturation rates in young premenarche patients. Fertil. Steril..

[17] Virant-Klun I., Leicht S., Hughes C., Krijgsveld J. (2016). Identification of maturation-specific proteins by single-cell proteomics of human oocytes. Mol. Cell. Proteom..

[18] Guo Y., Cai L., Liu X., Ma L., Zhang H., Wang B. (2022). Single-cell quantitative proteomic analysis of human oocyte maturation revealed high heterogeneity in *in vitro*–matured oocytes. Mol. Cell. Proteom..

[19] Galatidou S., Petelski A.A., Pujol A., Lattes K., Latorraca L.B., Fair T. (2024). Single-cell proteomics reveals decreased abundance of proteostasis and meiosis proteins in advanced maternal age oocytes. Mol. Hum. Reprod..

[20] Smith L.M., Kelleher N.L. (2013). Proteoform: a single term describing protein complexity. Nat. Methods.

[21] Toby T.K., Fornelli L., Kelleher N.L. (2016). Progress in top-down proteomics and the analysis of proteoforms. Annu. Rev. Anal. Chem..

[22] Johnson K.R., Gao Y., Greguš M., Ivanov A.R. (2022). On-Capillary cell lysis enables top-down proteomic analysis of single mammalian cells by CE-MS/MS. Anal. Chem..

[23] Su P., Hollas M.A.R., Butun F.A., Kanchustambham V.L., Rubakhin S., Ramani N. (2024). Single cell analysis of proteoforms. J. Proteome Res..

[24] Su P., Hollas M.A.R., Pla I., Rubakhin S., Butun F.A., Greer J.B. (2025). Proteoform profiling of endogenous single cells from rat hippocampus at scale. Nat. Biotechnol..

[25] Yang M., Hu H., Su P., Thomas P.M., Camarillo J.M., Greer J.B. (2022). Proteoform-selective imaging of tissues using mass spectrometry. Angew. Chem. Int. Ed..

[26] Yang M., Unsihuay D., Hu H., Meke F.N., Qu Z., Zhang Z.Y. (2023). Nano-DESI mass spectrometry imaging of proteoforms in biological tissues with high spatial resolution. Anal. Chem..

[27] Kafader J.O., Melani R.D., Durbin K.R., Ikwuagwu B., Early B.P., Fellers R.T. (2020). Multiplexed mass spectrometry of individual ions improves measurement of proteoforms and their complexes. Nat. Methods.

[28] Su P., McGee J.P., Durbin K.R., Hollas M.A.R., Yang M., Neumann E.K. (2022). Highly multiplexed, label-free proteoform imaging of tissues by individual ion mass spectrometry. Sci. Adv..

[29] McGee J.P., Su P., Durbin K.R., Hollas M.A.R., Bateman N.W., Maxwell G.L. (2023). Automated imaging and identification of proteoforms directly from ovarian cancer tissue. Nat. Commun..

[30] Su P., McGee J.P., Hollas M.A.R., Fellers R.T., Durbin K.R., Greer J.B. (2025). Standardized workflow for multiplexed charge detection mass spectrometry on Orbitrap analyzers. Nat. Protoc..

[31] Kafader J.O., Durbin K.R., Melani R.D., Des Soye B.J., Schachner L.F., Senko M.W. (2020). Individual ion mass spectrometry enhances the sensitivity and sequence coverage of top-down mass spectrometry. J. Proteome Res..

[32] Tsui E.L., Harris C.J., Rowell E.E., Laronda M.M. (2023). Human ovarian gross morphology and subanatomy across puberty: insights from tissue donated during fertility preservation. F. S. Rep..

[33] Amargant F., Zhou L.T., Yuan Y., Nahar A., Krisher R.L., Spate L.D. (2023). FGF2, LIF, and IGF1 (FLI) supplementation during human in vitro maturation enhances markers of gamete competence. Hum. Reprod..

[34] Wagner M., Yoshihara M., Douagi I., Damdimopoulos A., Panula S., Petropoulos S. (2020). Single-cell analysis of human ovarian cortex identifies distinct cell populations but no oogonial stem cells. Nat. Commun..

[35] Laskin J., Heath B.S., Roach P.J., Cazares L., Semmes O.J. (2012). Tissue imaging using nanospray desorption electrospray ionization mass spectrometry. Anal. Chem..

[36] Yin R., Burnum-Johnson K.E., Sun X., Dey S.K., Laskin J. (2019). High spatial resolution imaging of biological tissues using nanospray desorption electrospray ionization mass spectrometry. Nat. Protoc..

[37] Edwards N.J., Oberti M., Thangudu R.R., Cai S., McGarvey P.B., Jacob S. (2015). The CPTAC data portal: a resource for cancer proteomics research. J. Proteome Res..

[38] Ashburner M., Ball C.A., Blake J.A., Botstein D., Butler H., Cherry J.M. (2000). Gene ontology: tool for the unification of biology. Nat. Genet..

[39] Aleksander S.A., Balhoff J., Carbon S., Cherry J.M., Drabkin H.J., The Gene Ontology Consortium (2023). The gene ontology knowledgebase in 2023. Genetics.

[40] Thomas P.D., Ebert D., Muruganujan A., Mushayahama T., Albou L.P., Mi H. (2022). PANTHER: making genome-scale phylogenetics accessible to all. Protein Sci..

[41] Horn D.M., Zubarev R.A., McLafferty F.W. (2000). Automated reduction and interpretation of high resolution electrospray mass spectra of large molecules. J. Am. Soc. Mass. Spectrom..

[42] Milo R. (2013). What is the total number of protein molecules per cell volume? A call to rethink some published values. BioEssays.

[43] McDowell H., Vieco-Martí I., VanZanten M., Pant S., Kubo H., Saunders D.C. (2025). Digital analysis of ovarian tissue: generating a standardized method of follicle analysis. Biol. Reprod..

[44] Bebbere D., Albertini D.F., Coticchio G., Borini A., Ledda S. (2021). The subcortical maternal complex: emerging roles and novel perspectives. Mol. Hum. Reprod..

[45] Carmona U., Li L., Zhang L., Knez M. (2014). Ferritin light-chain subunits: key elements for the electron transfer across the protein cage. Chem. Commun..

[46] Rogalla T., Ehrnsperger M., Preville X. (1999). Regulation of Hsp27 oligomerization, chaperone function, and protective activity against oxidative stress/tumor necrosis factor alpha by phosphorylation. J. Biol. Chem..

[47] Demond H., Anvar Z., Jahromi B.N., Sparago A., Verma A., Davari M. (2019). A KHDC3L mutation resulting in recurrent hydatidiform mole causes genome-wide DNA methylation loss in oocytes and persistent imprinting defects post-fertilisation. Genome Med..

[48] Shibata Y., Voeltz G.K., Rapoport T.A. (2006). Rough sheets and smooth tubules. Cell.

[49] Kang X., Wang J., Yan L. (2023). Endoplasmic reticulum in oocytes: spatiotemporal distribution and function. J. Assist. Reprod. Genet..

[50] Vindry C., Marnef A., Broomhead H., Twyffels L., Ozgur S., Stoecklin G. (2017). Dual RNA processing roles of Pat1b via cytoplasmic Lsm1-7 and nuclear Lsm2-8 complexes. Cell. Rep.

[51] Bell M., Schreiner S., Damianov A., Reddy R., Bindereif A. (2002). P110, a novel human U6 snRNP protein and U4/U6 snRNP recycling factor. EMBO J..

[52] Gonzalez X.V., Almutlaq A., Gupta S.S. (2023). Systematic review of mRNA expression in human oocytes: understanding the molecular mechanisms underlying oocyte competence. J. Assist. Reprod. Genet..

[53] FitzHarris G., Marangos P., Carroll J. (2007). Changes in endoplasmic reticulum structure during mouse oocyte maturation are controlled by the cytoskeleton and cytoplasmic dynein. Dev. Biol..

[54] Auton A., Abecasis G.R., Altshuler D.M. (2015). A global reference for human genetic variation. Nature.

[55] Bebbere D., Abazari-Kia A., Nieddu S., Melis Murgia S., Albertini D.F., Ledda S. (2020). Subcortical maternal complex (SCMC) expression during folliculogenesis is affected by oocyte donor age in sheep. J. Assist. Reprod. Genet..

[56] Practice Committee of the American Society for Reproductive Medicine. (2019). Fertility preservation in patients undergoing gonadotoxic therapy or gonadectomy: a committee opinion. Fertil. Steril..

[57] Ethics Committee of the American Society for Reproductive Medicine. (2021). Disparities in access to effective treatment for infertility in the United States: an ethics committee opinion. Fertil. Steril..

[58] Driscoll G.L., Tyler J.P., Hangan J.T., Fisher P.R., Birdsall M.A., Knight D.C. (2000). A prospective, randomized, controlled, double-blind, double-dummy comparison of recombinant and urinary HCG for inducing oocyte maturation and follicular luteinization in ovarian stimulation. Hum. Reprod..

[59] Ito K., Takae S., Nakamura K., Furuyama S., Nakajima M., Suzuki Y. (2023). The study of the efficiency of in vitro maturation of ovarian tissue oocytes in pediatric patients. J. Assist. Reprod. Genet..

[60] Gilchrist R.B., Lane M., Thompson J.G. (2008). Oocyte-secreted factors: regulators of cumulus cell function and oocyte quality. Hum. Reprod. Update.

[61] Bonissone S., Gupta N., Romine M., Bradshaw R.A., Pevzner P.A. (2013). N-Terminal protein processing: a comparative proteogenomic analysis. Mol. Cell. Proteom..

[62] McTiernan N., Kjosås I., Arnesen T. (2025). Illuminating the impact of N-Terminal acetylation: from protein to physiology. Nat. Commun..

