# Supplementary material for: Probing Proteoform Heterogeneity From Single Human Oocytes
Source: Mol Cell Proteomics. 2026 Apr 9;25(5):101566. doi: 10.1016/j.mcpro.2026.101566 (PMC13194553; doi:10.1016/j.mcpro.2026.101566)
Supplement: Supplemental Material 1 [file mmc1.docx]

**Probing Human Oocyte Heterogeneity by Single-Cell Proteoform Imaging Mass Spectrometry**

Nickolas P. Fisher^1^, Vijaya Lakshmi Kanchustambham^1^, Elizabeth L. Tsui^4,5^, Chelsea Lock^1^, Tian Xu^1^, Hannah B. McDowell^2,3^, Indira Pla^1^, Diane C. Saunders^2,3^, Jared O. Kafader^1^, Monica M. Laronda^2,3,4*^, Neil L. Kelleher^1,5*^

**Table of Contents**

Supplemental Materials

**Figure S1.** Frequency bar plot of protein identification in denuded oocytes..............................S-2

**Figure S2.** Visual depiction of COCs vs. denuded oocytes.........................................................S-3

**Figure S3.** Brightfield images of COC after thawing, after washing, and after scPiMS.............S-4

**Figure S4.** PathScan images indicating slide area analyzed for scPiMS anlaysis.......................S-5

**Figure S5.** Comparison of THRASH features detected and proteoforms validated....................S-6

**Figure S6.** Distribution of validated proteoforms and THRASH detected proteoforms.............S-7

**Figure S7.** Comparison of total ions collected and charge assigned ions per oocyte..................S-8

**Figure S8.** PathScan images of intact and burst oocytes before scPiMS....................................S-9

**Figure S9.** Darkfield images of scPiMS probe sampling COC.................................................S-10

**Figure S10.** Brightfield images of four COCs used in the study...............................................S-11

**Figure S11.** Proteoform counts in oocytes and cumulus granulosa cells in COCs...................S-12

**Figure S12.** Interparticipant and intraparticipant similarity heatmaps......................................S-13

**Figure S13.** Number of LSM Protein Identifications by donor.................................................S-14

**Figure S14.** Example spectra of KHDC3 and OOEP proteoform landscapes...........................S-15

**Figure S15.** Proteoform mass error historgram.........................................................................S-16

**Supplementary Table 1.** Participant/donor demographic and clinical information.................S-17

**Supplementary Table 2.** List of proteoforms used for I^2^MS data calibration..........................S-18

**Figure S1.** Frequency bar plot of how frequently proteins were detected across the 24 denuded oocytes.


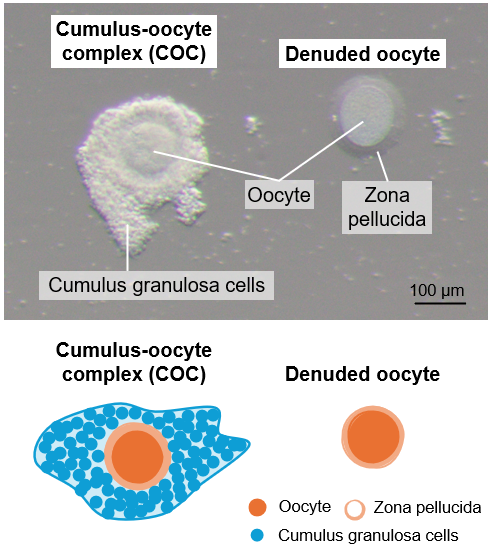


**Figure S2.** Visual depiction of a cumulus-oocyte complex (COC) vs. an oocyte that has been denuded to remove cumulus granulosa cells.


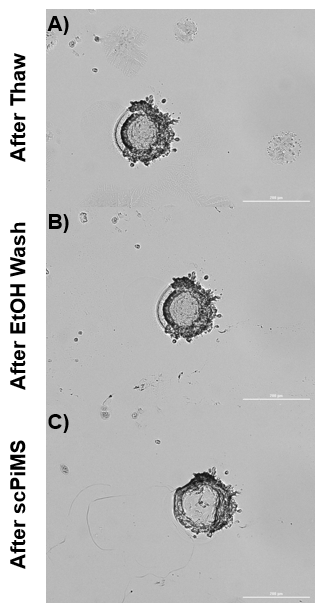


**Figure S3.** High-reslution brightfield microscopy images of COC **(A)** after thawing from -80 C storage, **(B)** after washing with increasing concentrations of ethanol, and **(C)** after scPiMS analysis.


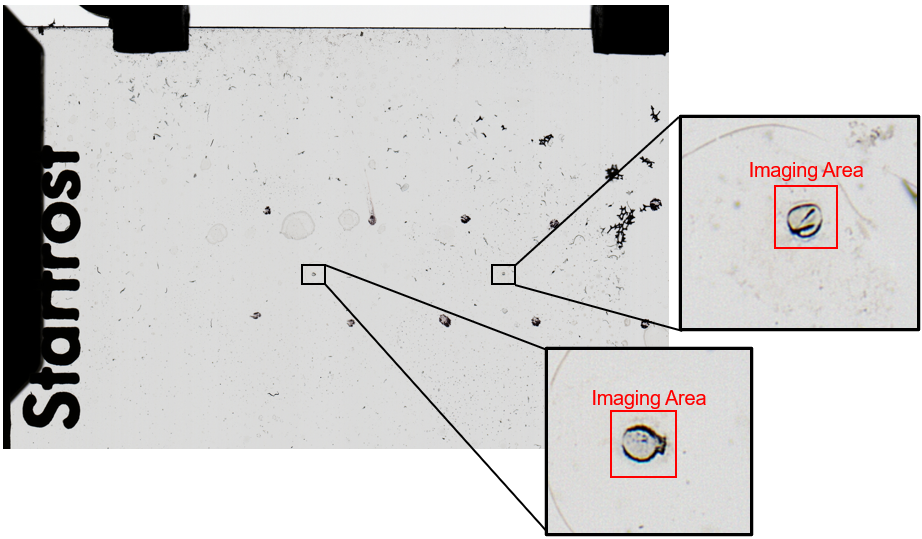


**Figure S4.** PathScan image of a slide containing oocytes and insets (red) corresponding to the part of the slide probed by scPiMS for single cell oocyte analysis.

**Figure S5.** Number of THRASH features detected in each oocyte mass spectrum compared to the number of proteoforms confidently assigned in each oocyte.


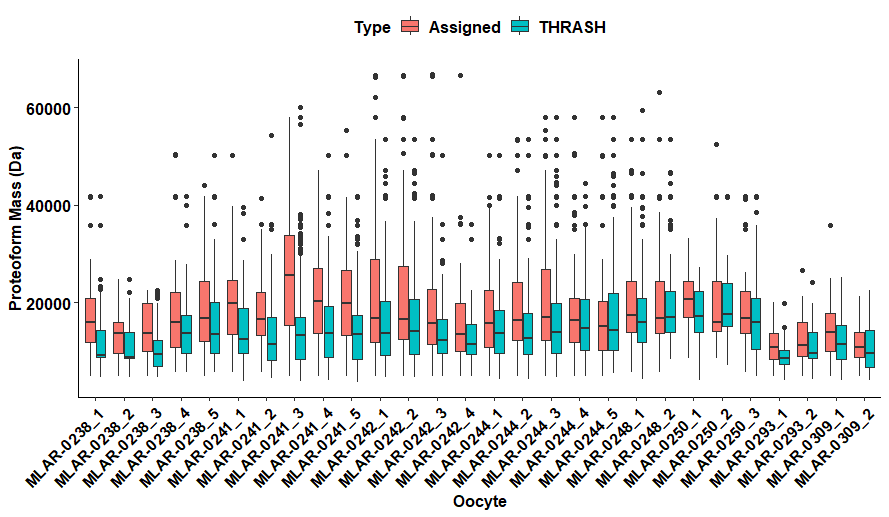


**Figure S6.** Distribution of identified and manually validated proteoforms (red) compared to the distribution of detected proteoforms via THRASH deconvolution (blue).

**Figure S7.** Number of total ions collected compared to the number of ions that were charged assigned and binned into a mass domain spectrum for each oocyte.


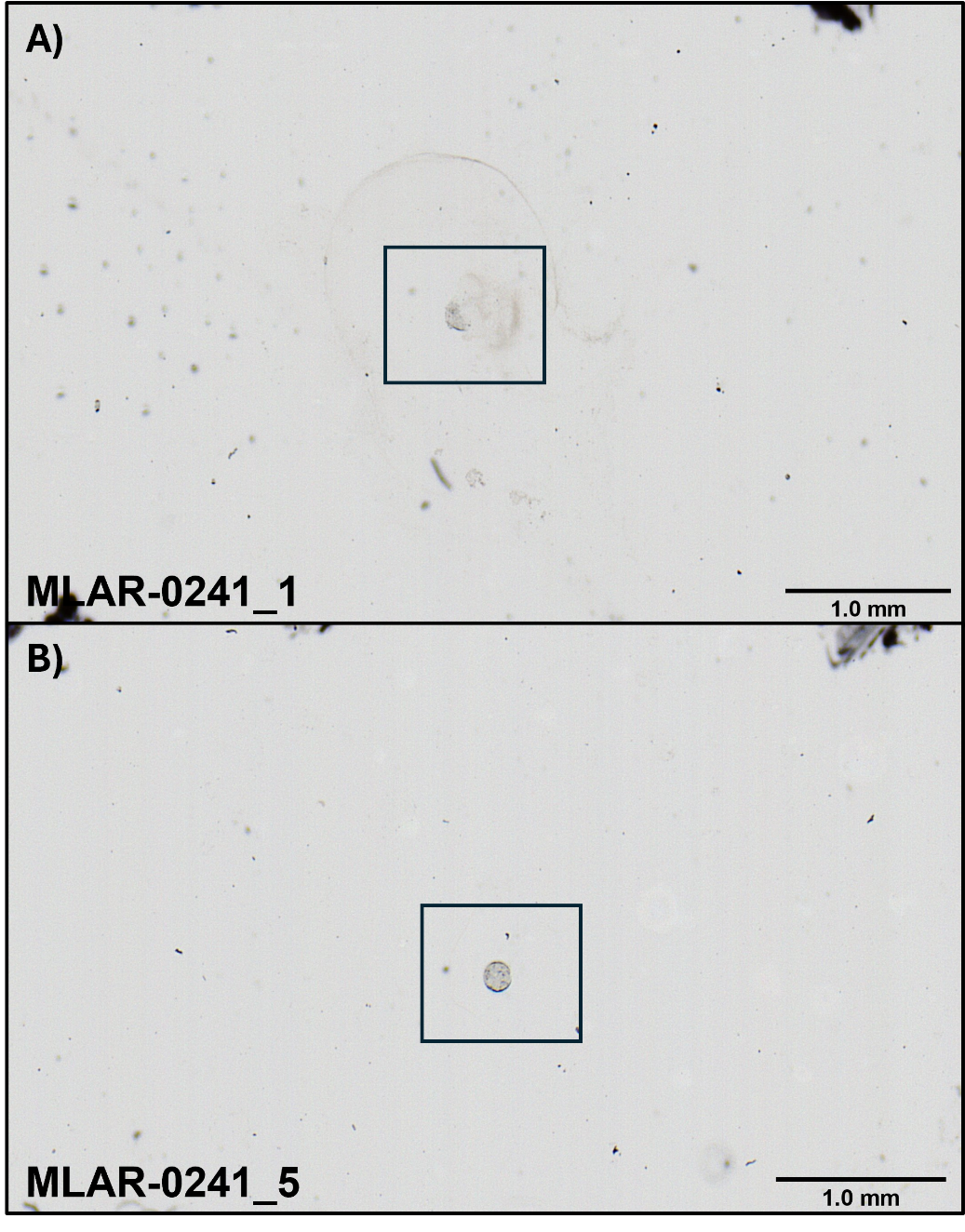


**Figure S8.** PathScan images of oocytes prior to scPiMS anlaysis with one burst oocyte **(A)** and one intact oocyte **(B)**.


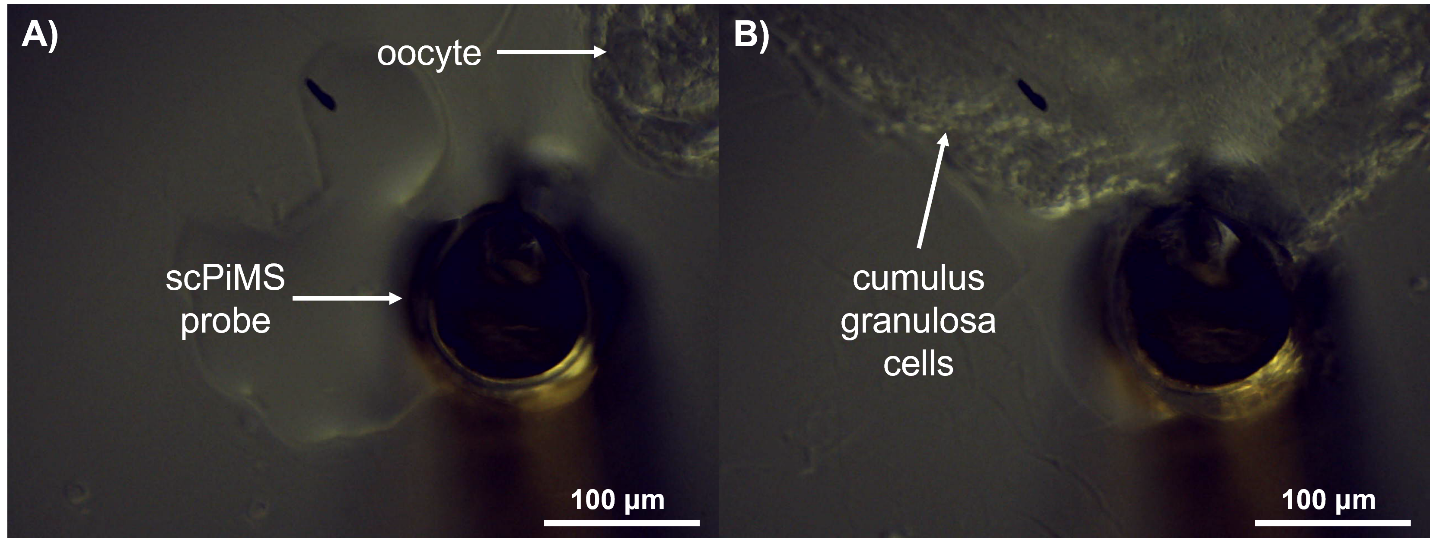


**Figure S9.** Darkfield microscope images from a Dino-Lite camera showing scPiMS probe in contact with the glass slide **(A)** and the scPiMS probe selectively sampling the oocyte from a COC **(B)**.


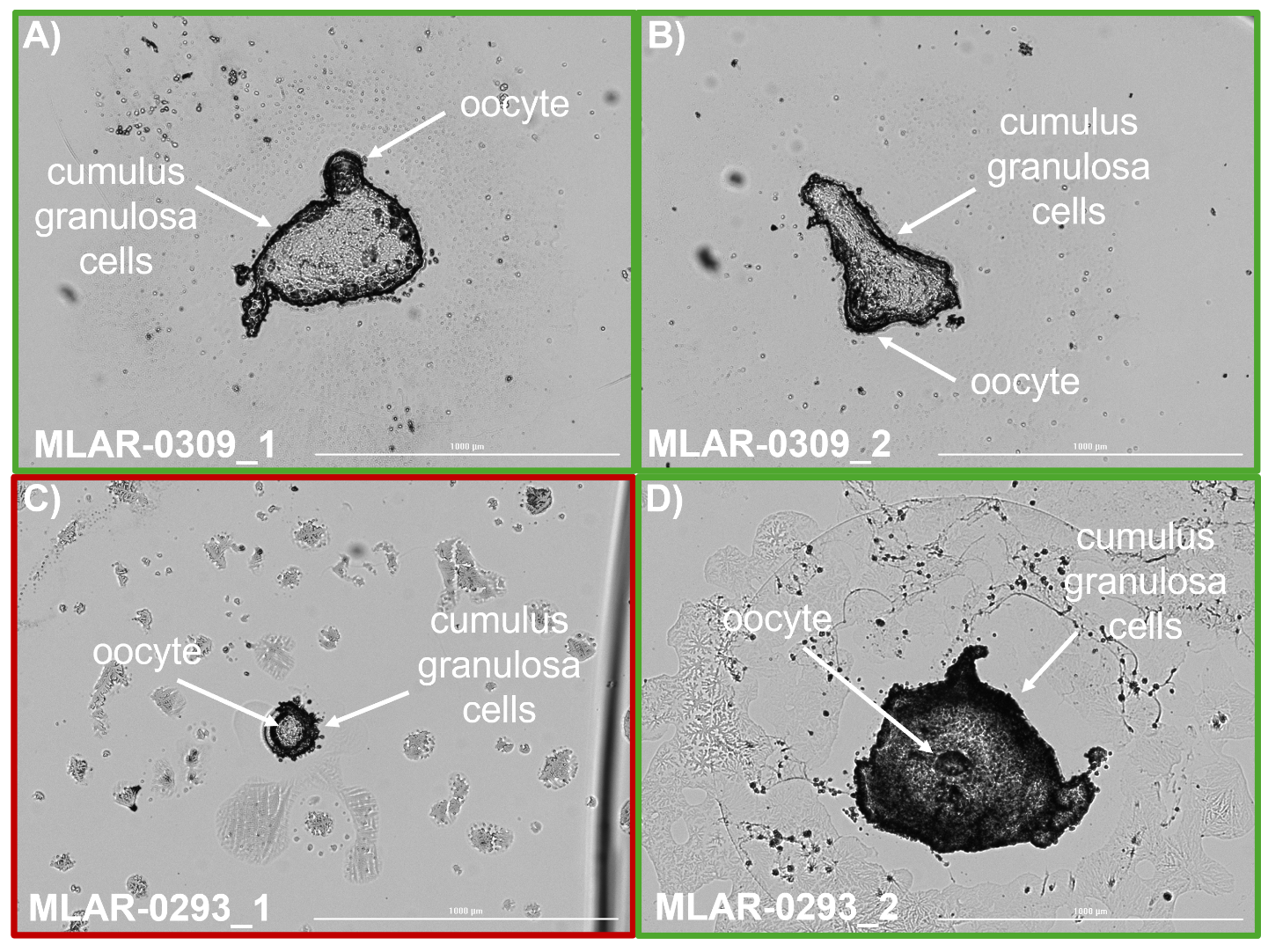


**Figure S10.** High resolution brightfield microscopy images of the 4 COCs used in this study. Three of the four COCs had large enough cumulus granulosa cell populations for selective sampling of these cells and the oocytes **(A, B, D)**. One COC with a small populaiton of cumulus granulosa cells that was not utilized for the selective sampling **(C)**.

**Figure S11.** Bar plot showing the quantity of proteoform IDs made for the oocytes (blue) and the cumulus granulosa cells (orange) from the same COC.


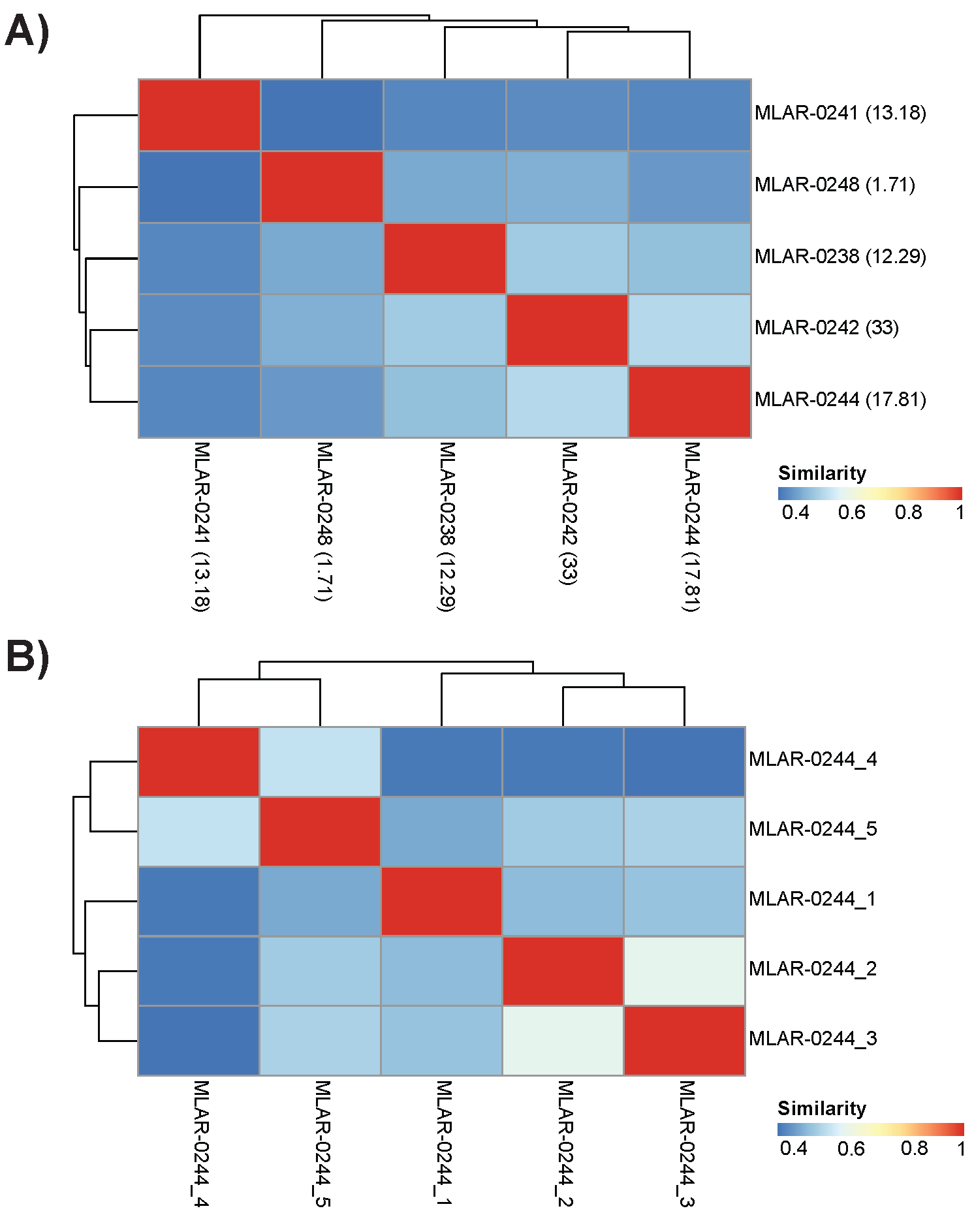


**Figure S12.** Heatmaps gauging interparticipant (A) and intraparticipant (B) oocyte proteome heterogeneity by identification similarity. Participant ages are given in parentheses.

**Figure S13.** Number of LSM proteins identified in 5 pateint compariosn, organized by age.

**
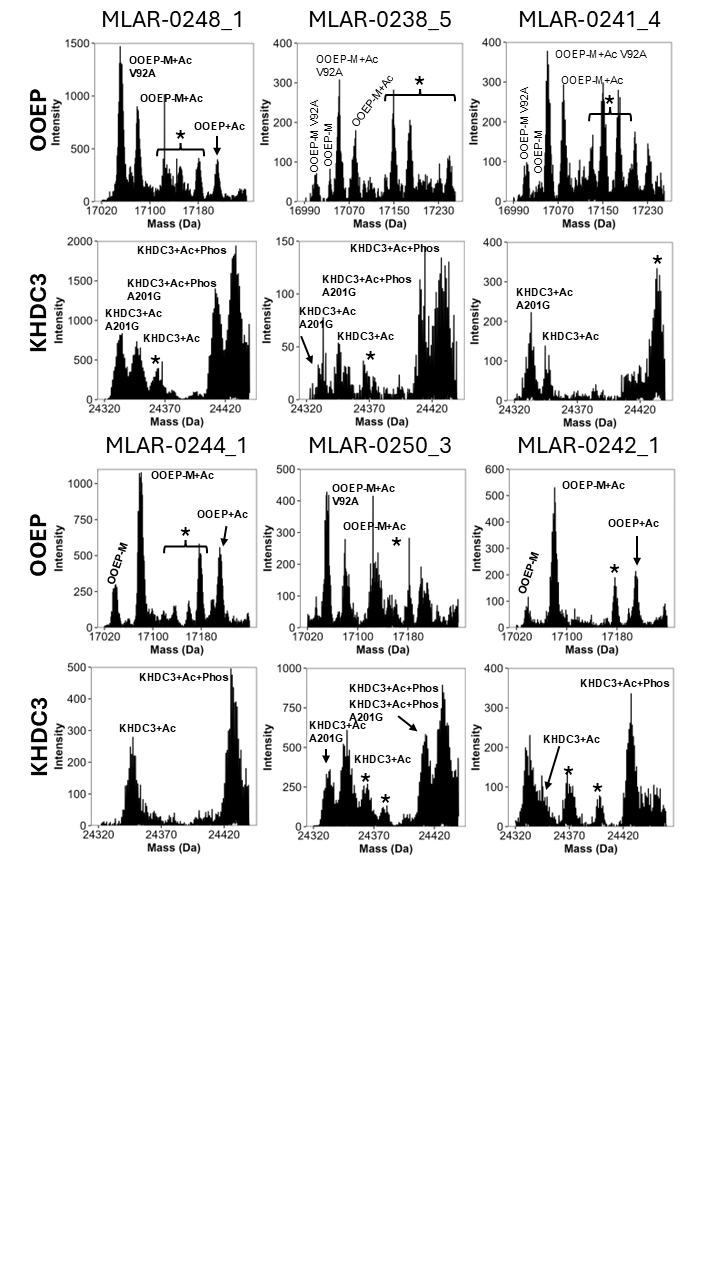
**

**Figure S14.** Example spectra of KHDC3 and OOEP proteoform landscapes from all 6 donors they were identified in. ***=**Unannotated


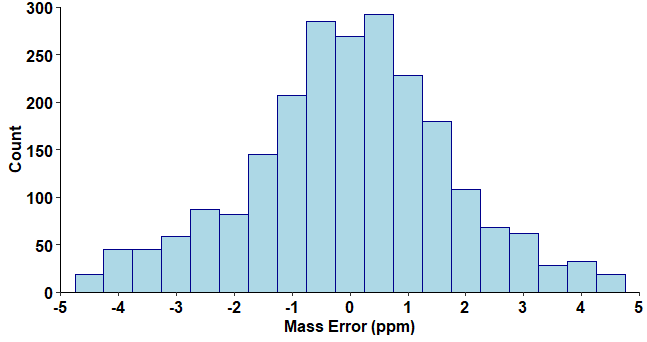


**Figure S15.** Proteoform mass error histogram binning proteoform identifications made in all oocytes by mass error (ppm) associated with each identification after spectral calibration.

**Supplemental Table 1.** Participant/donor demographic and clinical information

| **Deidentified ID** | **Age (years)** | **Tanner Stage** | **Pubertal Group** | **Diagnosis** | **Previous Chemo** | **Sample Type** | **# Oocytes** | **# COCs** | **Follicles (LC/MS-MS)** |
| --- | --- | --- | --- | --- | --- | --- | --- | --- | --- |
| MLAR-0248 | 1.71 | 1 | Prepubertal | Neuroblastoma | N | OTC media | 2 | - |  |
| MLAR-0309 | 4.08 | 1 | Prepubertal | Extracranial rhabdoid tumor | N | OTC media | - | 2 | - |
| MLAR-0238 | 12.29 | 3 | Postpubertal | Diamond blackfan anemia | N | OTC media | 5 | - | - |
| MLAR-0241 | 13.18 | 3 | Postpubertal | Alveolar rhabdomyosarcoma | Y | OTC media | 5 | - | - |
| MLAR-0293 | 13.81 | 4 | Postpubertal | Ewing sarcoma of the pelvis | N | OTC media | - | 2 | - |
| MLAR-0244 | 17.81 | 5 | Postpubertal | Hodgkin lymphoma | N | OTC media | 5 | - | - |
| MLAR-0250 | 19.93 | 5 | Postpubertal | Hodgkin lymphoma | N | OTC media | 3 | - | - |
| MLAR-0242 | 33.00 | N/A | Postpubertal | - | - | Whole ovary | 4 | - | - |
| MLAR-0074 | 8.44 | 2 | Postpubertal | Alveolar rhabdomyosarcoma | N | Cortical strips | - | - | Pooled |

**Supplemental Table 2.** List of proteoforms used for I^2^MS data calibration.

| **Proteoform** | **Modification** | **Theor. Mass (Da)** | **Mono m/z**  **(Da)** | **ppm**  **Error** | **Detected**  **Charges** |
| --- | --- | --- | --- | --- | --- |
| \|P56381\|ATP5E_HUMAN ATP synthase F(1) complex subunit epsilon, mitochondrial |  | 5645.07 | 5646.07 | 0.70 | 7-11 |
| \|P0CG47\|UBB_HUMAN Polyubiquitin-B |  | 8559.62 | 8560.62 | -0.18 | 10-14 |
| \|P0CG47\|UBB_HUMAN Polyubiquitin-B | Oxidation | 8575.61 | 8576.62 | 0.55 | 10-14 |
| \|P61604\|CH10_HUMAN 10kDa heat shock protein, mitochondrial | N. term. acetyl | 10835.84 | 10836.85 | -0.09 | 12-16 |
| \|P61604\|CH10_HUMAN 10kDa heat shock protein, mitochondrial | N. term. acetyl  Oxidation | 10851.84 | 10852.84 | 0.05 | 12-16 |
| \|P0DP23\|CALM1_HUMAN Calmodulin-1 | N. term acetyl  Trimethyl | 16779.85 | 16780.86 | -0.91 | 12-17 |
